# A life fulfilled: positively influencing physical activity in older adults – a systematic review and meta-ethnography

**DOI:** 10.1186/s12889-019-6624-5

**Published:** 2019-04-02

**Authors:** Gemma S. Morgan, Micky Willmott, Yoav Ben-Shlomo, Anne M. Haase, Rona M. Campbell

**Affiliations:** 1 Population Health Sciences, Bristol Medical School, University of Bristol, Bristol, BS8 2PS UK; 2 School of Policy Studies, University of Bristol, Bristol, BS8 1TZ UK

**Keywords:** Ageing, Physical activity, Qualitative, Meta-ethnography

## Abstract

**Background:**

Increasing physical activity in older adults remains a key public health priority in countries with a high burden of non-communicable disease, yet current interventions have failed to substantially increase population uptake with UK data suggesting that only half of 65–74 year olds report meeting recommended levels. The aim of this study was to conduct a systematic and inductive qualitative synthesis of the large body of qualitative research describing what influences physical activity at this age, and older adults’ experiences of physical activity.

**Methods:**

A qualitative meta-ethnography was chosen as the study design as this inductive approach can provide novel insights and generate new theory about physical activity and ageing. Papers were identified by searching electronic databases and key citations. Peer-reviewed primary qualitative studies and systematic reviews were included if they met the following inclusion criteria: community-dwelling participants aged 60 years or older or in the retirement transition period; reporting on leisure-time physical activity; utilising a rigorous qualitative methodology. A line of argument approach was employed to generate a theory about how older adults think and feel about physical activity.

**Results:**

Thirty-nine papers met the inclusion criteria and were synthesised. The emergent theory suggested transition to older age can challenge people’s sense of self and their role in life. Physical activity can help in regaining feelings of purpose, of being needed in collective group activity, and by creating habitual routine and structure to the day. In overcoming real and perceived barriers, and by taking up or sustaining physical activities, older adults can further build self-esteem all of which contributes to a fulfilling older age.

**Conclusion:**

Current failures to increase population levels of physical activity in older adults may be explained by an approach overly focused on the health benefits of activity. Insights from this study suggest we need to reframe our approach to consider the wider set of goals and aspirations which are of greater personal importance to older adults, and future interventions should focus on how physical activity can contribute to life satisfaction, sense of purpose, and sense of role fulfilment in older age.

**Trial registration:**

Registered prospectively on PROSPERO on 29th March 2013: CRD42013003796.

**Electronic supplementary material:**

The online version of this article (10.1186/s12889-019-6624-5) contains supplementary material, which is available to authorized users.

## Background

Whilst the scientific and technological progress in reducing premature mortality in high and middle-income countries is welcome, society must prepare for the significant changes this extension of life expectancy will bring to the way our populations are structured. There are vast health, social, and economic implications of an absolute and relative increase in the population of older adults, especially if these individuals are in poor states of health with high levels of frailty and disability [1]. To ameliorate the associated burden on health and social care services it is critical to identify ways of supporting older adults to enjoy active, independent, and happy lives for as long as possible. Emerging evidence suggests that physical activity in later life may prevent, or at least delay, the onset of age-related functional impairment [2–4]. There is a wealth of literature describing different interventions with older adults, of varying delivery method and varied methodological rigour of evaluation. Several small and two large randomised controlled trials (RCTs) [5–12] show some evidence of effectiveness, however, these do not provide definitive evidence because of small sample sizes; self-selecting populations; short follow-up periods; small, inconsistent, or absent effects; or because the interventions are of an intensity and cost that would impede routine implementation. The cited studies [5–12] highlight how research into interventions to increase physical activity amongst older adults has not delivered very impressive results overall. Moreover, there is emerging evidence that the design of the built environment is associated with physical activity levels in older adults [13] and recent research from the United States (US) has highlighted the role of social capital in leisure-time physical activity in those aged over 65 years [14]. New approaches may therefore be required to ensure that behavioural interventions address the principal drivers that affect older adults’ behaviour.

A deeper understanding of what physical activity signifies to older adults would enable the development of interventions that acknowledge and address the physical, psychological, and social factors that influence their activity. Qualitative studies can be of considerable value in answering such questions. There is a very large and disparate body of qualitative research in this field to draw on, and there is a need for this to be quality-assessed and assimilated. Integrating or synthesising qualitative research findings can produce new insights not evident in single studies and can generate new theory which can be used to inform the development of new interventions. For example, a meta-ethnography of qualitative research revealed new insights into how patient experiences are shaped by the processes of decision-making and meaning-making. The meta-ethnography indicated ways in which communication between doctors and patients may be facilitated in terms of antidepressant concordance [15]. Theory is important in developing new interventions as evidence suggests that public health or behavioural interventions based on behaviour-change theory are more likely to be effective than those not underpinned by theory [16–18].

Whilst there have been several robust systematic quantitative reviews of the effectiveness of physical activity interventions for older adults, existing qualitative syntheses have been limited in scope, for example focusing only on those of South Asian heritage [19], those with a diagnosis of arthritis [20], those participating in intervention studies [21], and those in transition to retirement [22]. Findings from reviews focused only on these groups may not be generalisable to the wider population of older adults, which will include those who are long retired and those who have never worked. One review, which did not focus on a specific group, contained over a hundred qualitative studies [23]. In-depth and inductive analysis would be impracticable with such a large and wide-ranging set of studies and analysis in this paper was limited to content analysis, which is recognised as a quantitative approach [24]. Whilst that paper provides a useful aggregative summary of the field, a more interpretive synthesis technique can utilise all the rich conceptual data available in individual studies and generate new concepts and theory. Meta-ethnography is one such technique and involves reconceptualisation of the original papers by considering the set of papers as a whole, greater than the sum of their parts [25]. This is achieved by systematically comparing study concepts to identify new overarching concepts and new interpretations [26]. The results of a meta-ethnography may therefore differ considerably from the data contained within the constituent papers as it is interpretive rather than aggregative [25]. Whilst aggregative methods aim to collate, amass, and deduce findings as a way of summarising data, interpretive research is focused on building meaning through integrating the data. In interpretive syntheses, theories and insights are developed through the author’s interpretation of the combined findings of the original papers. Barnett-Page and Thomas provide further explanation of the methodologies for synthesising qualitative studies including the spectrum of interpretive and non-interpretive approaches [27].

We aimed to undertake a thorough and comprehensive meta-ethnographic synthesis of high-quality qualitative studies on older adults and physical activity in order to generate theoretical insights which could be used to enhance intervention development in this field.

## Methods

### Eligibility criteria

Papers were included if they were peer-reviewed and reported primary qualitative or mixed methods studies, including systematic reviews. Only papers which focused on physical activity and included community-dwelling participants aged 60 years or over, or those in the retirement transition period, were included. Studies reporting subgroups containing adults in this age range were included if the older adults were analysed and reported separately. Physical activity was considered to include leisure-time physical activity (defined as activity not conducted as part of occupational work or medical rehabilitation), active pastimes (defined as activities that involved physical activity and were undertaken for enjoyment), and sports. Papers were excluded if the population was recruited because of a specific disease or health condition, participants had been selected because they were “master athletes”, if physical activity was only a minor component of a wider set of behaviours investigated, and if the physical activity was only in the context of a clinical rehabilitation programme or as part of a research evaluation of an intervention. Studies reporting only superficial, quantitative, or other deductive analytical techniques such as content analysis or those using pre-set frameworks were excluded because these papers would not have had the richness and depth of concept obtained from studies using inductive approaches. Quantitative approaches include counting the frequency with which certain concepts appear in the data, and deductive analytical approaches are often “quasi-quantitative” in that they start with a hypothesis and use the data to refute or confirm this, rather than being led by the dataset itself to drive the theory. Inductive approaches are more fitting for qualitative research where meaning and understanding are sought from the data. Only papers published in English were included.

### Search strategy

**Fig. 1:**
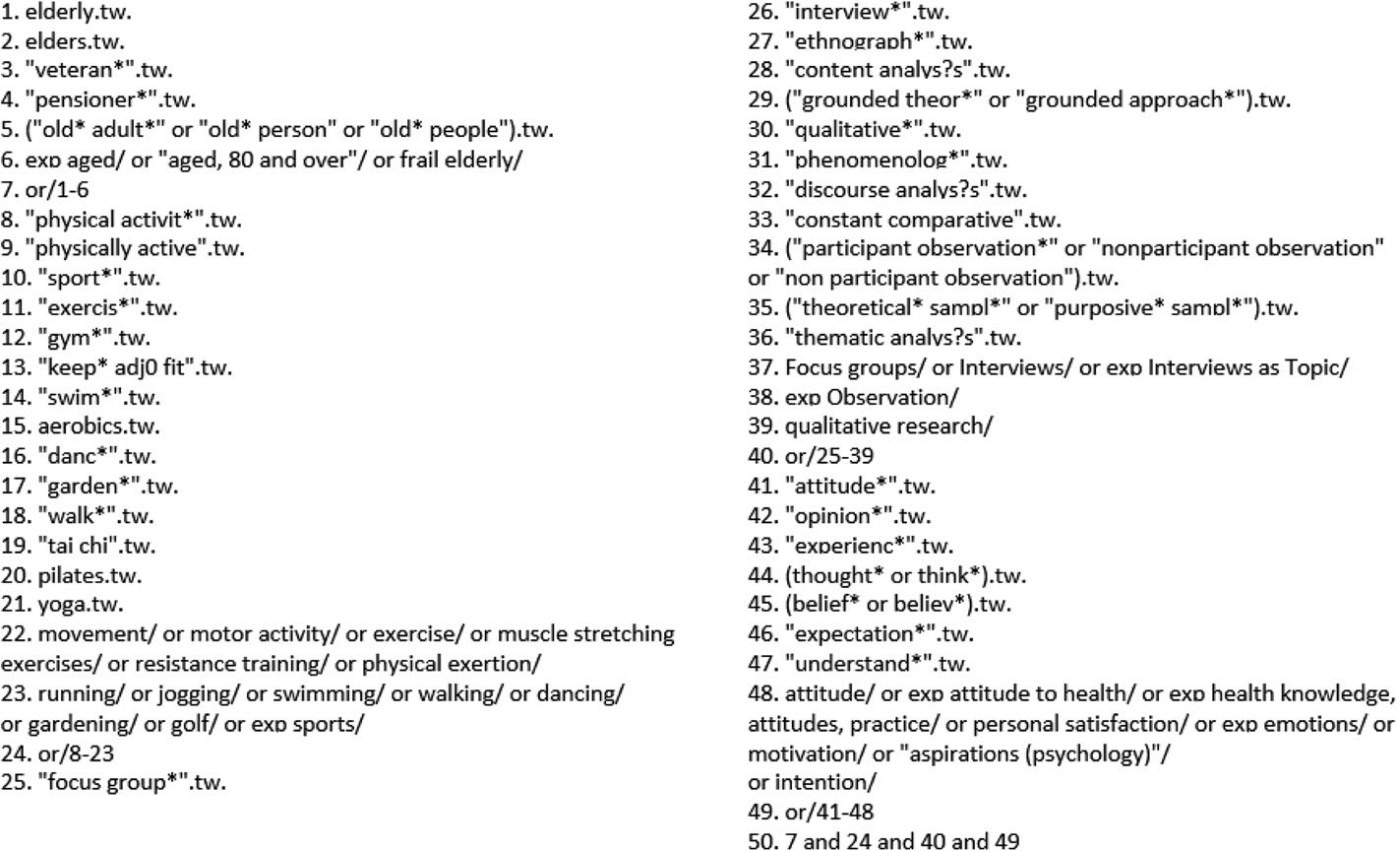
Search terms used in Medline

The following electronic databases were searched in January 2013 to identify all potentially relevant titles: Ovid Medline; Ovid Embase; Ovid PscyhINFO; CINAHL; Web of Science. Search strategies were devised to ensure maximum sensitivity with reasonable specificity (see Fig. 1 for an example set of search terms used in Medline; the full search strategy is available on request). Sources of “grey literature”, for example websites run by voluntary sector organisations that promote healthy ageing, were also searched for reference to peer-reviewed papers. All retrieved studies from the main searches were imported into EPPI-Reviewer 4 [28] review manager, which was used to record decisions at screening and appraisal stages. In an update in October 2016, the same search strategy was run in the same databases, covering the period 01/01/13–20/10/16.

### Quality appraisal

The role of quality appraisal in qualitative syntheses is debated [29], however if findings of qualitative syntheses are to be used to influence health policy then the findings of individual papers must be trustworthy and have integrity. A modified version of the Dixon-Woods quality assessment framework [30] was used by GSM and MW to independently appraise each paper; where there was disagreement this was resolved by discussion. Papers were categorised as “good enough” or “fatally flawed” with the latter excluded from further analysis. The focus of the quality assessment was on both conceptual clarity (i.e. whether the paper reports concepts and themes resulting from in-depth theoretical analysis) and interpretive rigour (i.e. whether it was possible to be convinced that the findings had been reached from a robust and inductive examination of primary qualitative data). The framework developed by Toye et al. [30] was used to make these decisions, and includes assessment questions such as: whether the concepts are clear and translatable; whether the authors demonstrate interpretive rigour in the analysis; whether the authors describe contradictory data; and what the ethical context of the study is. Papers classed as “fatally flawed” were those which the authors believed contained insufficient detail to be deemed trustworthy.

### Data extraction

A meta-ethnography involves extraction and translation of second-order constructs (the interpretations of the original papers’ authors) to generate third-order constructs. Third-order constructs are novel and innovative, and should reveal something about the whole field that is not evident from simply reading the original papers [15, 31].

#### Extracting and translating second-order constructs

Second-order constructs were extracted by the first author by line-by-line coding of all text under the headings of “Results” or “Findings”. Free second-order construct codes were generated de novo to capture the findings described in the text. As new codes were generated, they were juxtaposed with existing codes and a process of refinement, re-classification, and grouping was undertaken. A purposive sample of papers, that is those felt to contribute the greatest to the emerging findings, were double-coded by MW who was blinded to the code set created by GSM. Double-coding was employed to broaden interpretations rather than to confirm or refute the original coding, given that precise agreement is neither likely nor necessary in meta-ethnography [31]. Extracted second-order constructs were then tabulated, systematically considered according to features of the population studied (e.g. gender, age, ethnicity, culture), and translated [15]. The set of translated codes was discussed with co-authors to ensure the data were plausible and comprehensive. A conceptual map was devised, cross-referencing and linking second-order constructs and recording which papers the construct appeared in. Contextual detail was preserved during this mapping phase to allow exploration of relationships between papers and constructs.

#### Synthesis of translations

The conceptual map and tabulated translations were then examined as a whole by GSM, to identify how key concepts could be connected, contrasted, and synthesised to create a “line of argument”. A “line of argument” is one of several recognised approaches to the final stage of meta-ethnography, and is best defined as “… *to offer a fuller account of the phenomenon by arranging the studies’ metaphors in some order … to construct an argument about what the set of ethnographies say”* [32]. To facilitate the synthesis, translated concepts were arranged in groups and a “meta-conceptual level” of third-order concepts was created and used to generate the emergent line of argument.

Second-order constructs that appeared to refute or contradict the line of argument were considered to make sense of how they fitted into the emergent theory. Not all second-order constructs were incorporated into the line of argument synthesis. Some papers, most often those of weaker quality, provided conceptually poor data and only rather prosaic second-order constructs could be extracted. Whilst all second-order constructs were considered and translated, those not adding to the emerging theory or contributing to the development of third-order constructs were dropped from the final step of synthesis.

## Results

### Search and screening results

**Fig. 2:**
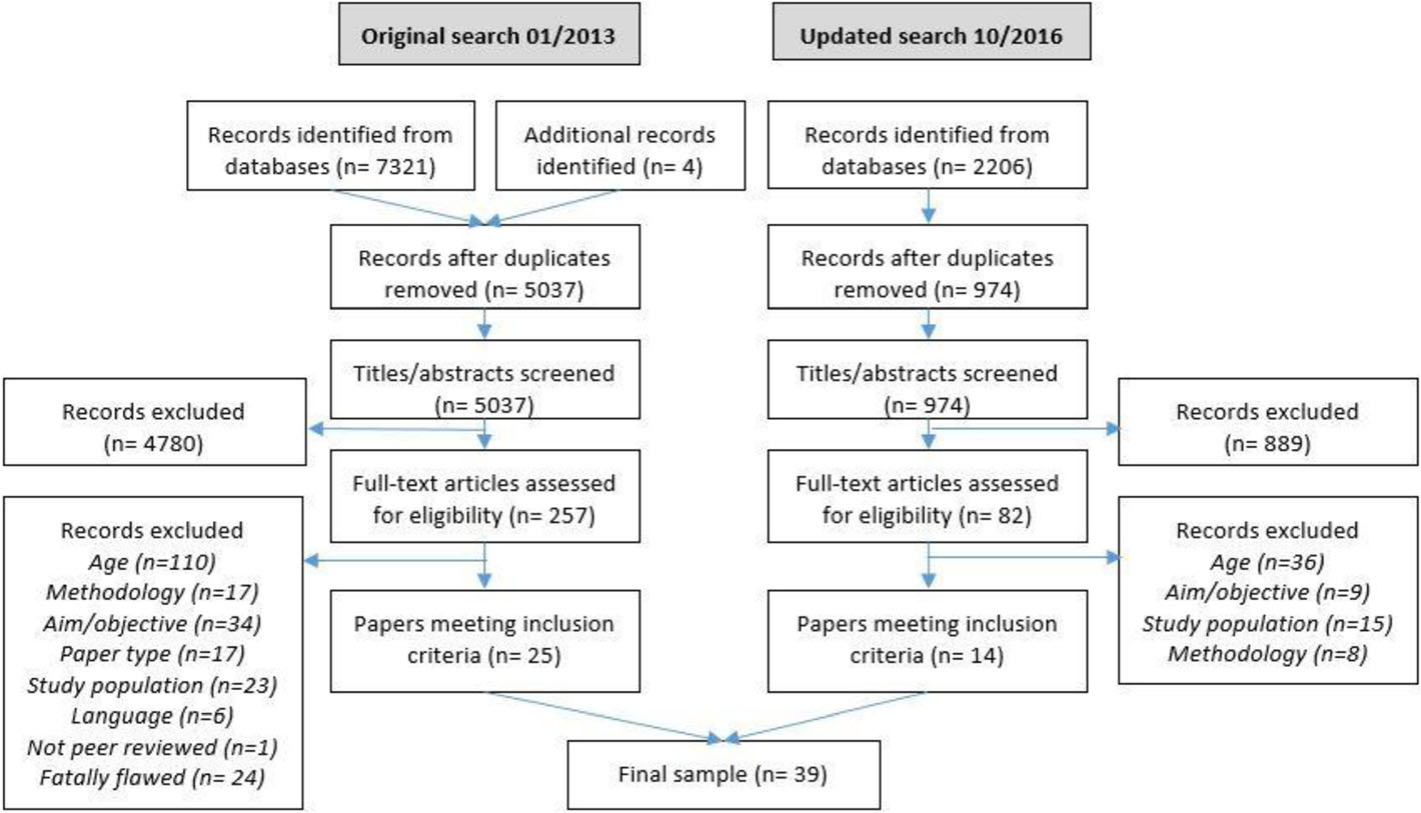
PRISMA diagram

Following removal of duplicated records, 5037 title and abstracts were screened by the first author with a random subset (> 10%) double-screened by a co-author; assessment revealed strong agreement (ĸ = 0.74). After the searches were repeated in October 2016 an additional 974 title and abstracts were screened. A total of 338 papers were subject to full-text review. Following quality appraisal, a final sample of 39 papers were included in the synthesis (25 from the original search and 14 from the updated search). The PRISMA diagram is shown in Fig. 2.

### Description of papers

The final sample included thirty-seven primary qualitative papers and two systematic reviews. The characteristics of included papers are listed in Additional file 1: Table S1. Most of the primary studies reported using semi-structured interviews (*n* = 32), other methodologies included focus groups (*n* = 9), ethnography or observations (*n* = 5), and photo-elicitation (*n* = 2); overall number exceeds 37 as many studies used multiple methodologies. The earliest paper included in the sample was published in 1995; 26/39 were published in this decade. None of the included papers focused only on men and five papers had female-only populations. Eleven (28%) of the study populations were from the United Kingdom (UK) and eleven from the US; only three papers studied populations not from a high-income country and these were Iran (*n* = 2) and China (*n* = 1), both upper-middle-income countries according to the World Bank [33]. Most study samples included a range of ages from 60 to 90 years with a small number (*n* = 6) including only participants over 70 years. Three papers and one review included some participants who were aged under 60 years; these were all studies about the retirement transition period.

### Second- and third-order constructs

Table 1 lists the key translated second-order constructs retrieved from the papers and tabulated alongside the summary third-order constructs. Five key third-order constructs were interpretively synthesised from the extracted data and were used to generate a line of argument theory:

**Table 1:** Translated second-order and third-order constructs synthesised and influencing emerging theory

Translated second-order constructs	References	Summary of third-order construct
Life events leading to a change in circumstances or role and triggering reflection on the new stage of life	[22, 34, 37, 38, 40–42, 54, 69, 72]	**How physical activity fits in with transition events in ageing** Major life events such as bereavement, retirement, end of caring role for children or grandchild can act as triggers leading to an awareness of the ageing body. Any negative feelings about ageing may be compounded by societal attitudes, stereotypes, and the expectations of others such as family. However, recognition of this new phase of life can be positive and lead to renewed motivation to stay youthful, maintain energy levels and vitality, and look after the physical body to maintain independence. For those with established physical activity habits, continuing lifelong patterns can facilitate this transition.
Reflection on lifelong patterns and past experiences with physical activity, including establishment of healthy habits early in life	[22, 23, 34, 36, 39–41, 46–48, 59, 62, 63, 69]	
Personal awareness of the ageing body and functional limitations to independence. Physical activity as a way of preventing ageing, a source of rejuvenation and youthfulness, giving new energy	[34, 37, 39, 41–46, 49–51, 53, 54, 57, 60, 61, 67]	
Societal attitudes and stereotypes about ageing and physical activity	[40, 41, 44]	
Physical appearance in ageing and effects of physical activity on image	[41, 44, 46, 54, 67]	
Routine and discipline	[22, 36, 37, 41–43, 53–56, 62, 63]	**The role of physical activity in older adults’ sense of purpose and self-belief** Physical activity forms an important part of an older adult’s daily routine and is conceptualised as a personal responsibility requiring discipline and taken seriously. In this way physical activity contributes to older adults’ role identity and sense of purpose. Being physically active in older age can provide a sense of personal achievement and satisfaction in mastering a new activity or maintaining an active life despite the challenges of ageing. Physical activity can also provide individuals with satisfaction and personal gratification from a busy life filled with reasons to get up and out of the home.
Undertaking physical activity is a responsibility, like a job, and gives a sense of purpose	[36, 37, 44, 48–51, 55, 62]	
Self-identity, self-esteem / confidence, and personal development	[44, 46, 58, 69]	
Self-belief, personal satisfaction, and feelings of mastery in overcoming physical activity challenges	[36, 44, 49, 53, 56–58]	
Being busy, having a reason and purpose to leave the house	[38, 44, 47–49, 69]	
Taking control over physical activity decisions	[34, 38, 39, 44, 53, 57]	
Belonging, togetherness	[36, 41, 42, 44, 49, 51, 53, 55]	**Physical activity creates and strengthens feelings of togetherness, community, and belonging** Physical activity provides access to social contact with others, leading to a sense of togetherness, of community, and of belonging to a group. Identifying as a member of a group in this way enhances self-esteem. The structural and functional support, and intimate or sexual relationships associated with shared physical activity further add to feelings of being needed by others. However family concerns and self-comparisons within social networks may impact negatively on physical activity behaviour.
Meeting people socially brings companionship and support	[22, 23, 36, 38, 41, 42, 46, 49–51, 53, 55, 56, 58–61, 63–65, 67–69]	
Facilitation of intimate and romantic relationships through physical activity	[41, 54]	
Competitiveness and fear of others being better	[23, 50, 51, 62]	
Social and family network inhibiting PA, lack of encouragement, differing views between spouses	[37, 62, 66]	
Physical health benefits	[22, 23, 39, 40, 44–46, 49, 56, 58–61, 63–69, 71]	**Physical activity effects on physical, emotional, and cognitive health** Health benefits of physical activity are widely accepted and for older adults the value of physical activity in contributing to positive wellbeing through enjoyment, laughter, and pleasure is important. Moreover, older adults are aware of the overlap in physical activity, “getting out and about”, and social contact and the positive effects of these on their mental health. Cognitive functions such as maintaining an “active mind” were also felt to be important benefits of an active lifestyle, alongside an appreciation of enriching and aesthetic components of activity including music and nature.
Pleasure, humour, happiness, fun, self-gratification, mental health benefits, wellbeing	[22, 38, 41, 42, 45, 46, 49, 50, 54, 56, 58, 61, 65, 67]	
Maintaining an active brain, mental stimulation	[41, 42, 44, 51]	
Nature, environment, outdoor activity	[38, 57, 63–65, 69, 72]	
Music, aesthetics, utilising cultural capital developed over lifetime	[41]	
Practical barriers (access, facilities, cost, time limitation, weather) and practical support to overcome this from family	[23, 37, 38, 43, 46, 50, 53, 60–64, 66, 68]	**Barriers to physical activity** Older adults experience external and logistical barriers to physical activity however these may be overcome with support from others including family. Internal barriers, such as fear of injury and pain, also exist for many and support from respected healthcare professionals can be helpful.
Fear of injury, pain, safety, health limitations	[22, 23, 40, 44, 48, 50, 53, 58–61, 64–66, 68, 69, 72, 89]	
Healthcare professionals	[46, 58, 89]	

#### How physical activity fits in with transition events in ageing

Major life events such as ill health [34], retirement [35–37], completion of the caring role for children or grandchildren [38], and bereavement [38] can act as triggers leading to an awareness of ageing and the “body finitude” [39], compounded by negative societal attitudes of older adults and their physical activity: “*Young girls in their twenties can be quite rude, but you can still snap back at them if you have a keen mind … It’s just self-absorbed people that dump you in a pile, you’re old, we can’t be bothered with that sort of attitude”* [40]*.* Physical activity is therefore used to reject stereotypes of ageing [41] and to view ageing through a positive “graceful” lens [42]. Rejection of such stereotypes can occur through taking up new activity in retirement [37, 43–45] [*“I don’t want to be a little old lady sitting around in a chair”* [46]], and this can be invigorating and revitalising, a source of inspiration and energy. Lifelong patterns of activity are important and for some older adults physical activity may provide an opportunity to revisit happy memories of their youth*: “One of the good things about this is that you become a teenager again …*” [41].

#### The role of physical activity in older adults’ sense of purpose and self-belief

Physical activity can bolster self-esteem and strengthen self-identity by adding routines and new roles in ageing [38], by fulfilling a need to keep busy [47, 48] and by providing a sense of purpose [49–54] and structure [37, 54] to the lives of older adults: “*I get up the same time as I did when I went to work. I do exactly the same thing the only difference is I go out … to the [swimming pool] and come back but I keep that momentum”* [37]. In this sense physical activity may be conceptualised as a responsibility, a duty requiring self-discipline [42, 54] and likened to occupational work [44, 48, 55]: *“It’s just like a job to me*” [55]. Physical activity routines are deemed important by older adults for facilitating sustained activity [36, 43, 56] and for the associated sense of exercising personal control that these routines can provide [34, 38, 39, 44, 53, 57] at a time where autonomy and independence may become reduced. The stability or continuity that regular and habitual physical activity affords may counter the changes inherent with ageing such as physical and functional decline, disability, and death of loved ones [41]. The papers revealed how taking up physical activity in later life can provide older adults with feelings of mastery [58], self-belief [53], and satisfaction in overcoming personal challenge [35, 36, 44, 49, 56] and simply “getting out and about” and staying busy [38, 44, 47–49, 52] may bolster self-esteem and help avoid feelings of helplessness and futility.

#### Physical activity creates and strengthens feelings of togetherness, community, and belonging

The use of physical activity as an opportunity to increase desired social contact with others [22, 23, 35, 38, 42, 49–52, 55, 57–61], in contributing to intimate relationships [41], and as a source of support [19, 50] were also key concepts reported in the papers. The value of being part of a group or community seemed to be linked to a sense of collective togetherness and belonging [41, 42, 44, 53, 55], which can lead to a sense of responsibility as members of the group rely on the attendance of others for support [49, 56]: “*People will notice and it makes you feel nice … it makes you feel … a bit special if people notice that you are not there”* [36]*.* Group membership per se was perceived to be of value to older adults [36, 49, 57] and was linked to establishment of health-benefitting routines [57, 19] though also held potentially adverse impacts including feelings of not being accepted or fitting in [22, 36] and fear of comparisons with others who were more able [23, 50].

The role of family in physical activity experiences in older age can be complex and discordant: practical support, understanding, and enjoyment of doing things with loved ones [*“Even when we’re away on holiday together you know, we go to places and climb hills and walk …*” [62, 19] can be valued [41, 46, 49, 61, 63, 64] but the desire for personal space [62], negative attitudes [37], and being “nagged” by spouses [62] may be detrimental, as can caregiving responsibilities [51, 65].

#### Physical activity effects on physical, emotional, and cognitive health

Pleasure derived from a feeling of belonging, a purposeful self-identity, and a habitual routine compliments the pleasure that older adults derive directly from the physical activity itself [54, 58]: *“You don’t know what goes on behind these doors – if only the others could see how we are enjoying ourselves”* [41]*.* For many active older adults, physical activity is associated with feelings of joy [65], laughter [42], and fun, along with perceived improvements to mental health [35, 38, 41, 45, 46, 50, 51, 56, 61] and keeping one’s mind active [39, 41, 44, 51].

The physical health benefits of physical activity are well established and widely acknowledged ( [22, 23, 39, 40, 44–46, 49, 56, 58–61, 63–71] yet physical activity also provides older adults with sensual enjoyment by exposure to music, smells, and touch not otherwise experienced in daily life [41, 54]: “*Being in such close contact with my husband when we’re dancing is wonderful. I always become very aware of his aftershave and I always like to wear nice perfume”* [54]*.* Similarly, for older adults who find pleasure from being outside and close to nature, physical activity can provide an opportunity to explore this [52, 57, 64, 65, 72]: “*… I like just looking at the sky you know, it is wonderful, the big skies are so beautiful. I walk through the fields there. I go for the skies alone”* [72]. Pleasure and enjoyment through physical activity appeared not to be any less important to older adults than to younger people; an awareness of mortality associated with ageing may indeed heighten the personal value placed on feelings of pleasure.

#### Disincentives and barriers to physical activity

External barriers to older adults’ physical activity are real and include time [46, 66] weather [63], cost [37], and structural barriers in the environment [50, 63]: *“You are naturally afraid of [walking on] ice, afraid of falling …*. *You take more care, don’t walk as energetically.”* [63] In addition, older adults can perceive that they might be “doing harm” [65] or overdoing it, causing pain or damage [73]. Mechanisms for overcoming these barriers includes trustworthy advice from facility staff [53] and healthcare professionals [46]. Support from spouses may be a way of overcoming motivational barriers: “*… yes I did slow down for about six months, and I would say that I am more sedentary now than I would like to be, but, you know, my wife says I’ve got to switch that telly off more often and get out and do something …*” [62].

### Line of argument

**Fig. 3:**
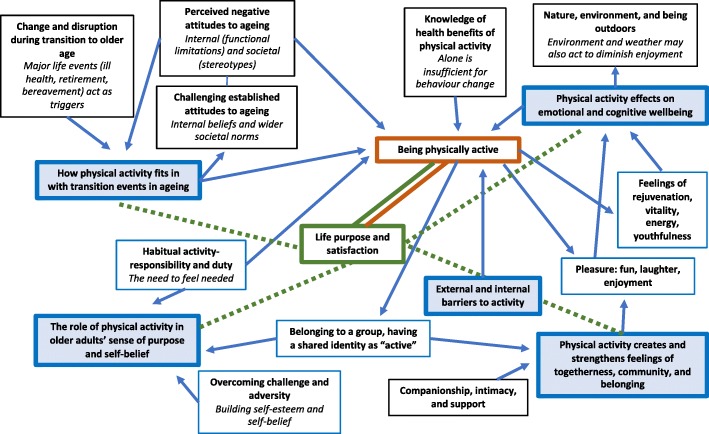
Line of argument: what physical activity means to older adults

Using a line of argument approach, our meta-ethnographic synthesis generated a theory of how physical activity fits into the ageing period, as depicted in Fig. 3. This argument asserts that accepting the transition to older age, which may be triggered by an acute event or reached gradually over time, can be a time of turmoil. The realisation that one’s role in life and personal identity is changing can leave a sense of loss: of structure, purpose, and control over life; of physical function; and of loved ones. Physical activity can play a part in regaining feelings of purpose and of being needed by engaging older adults in group activity where they feel a sense of responsibility to others, and by creating habitual routine and structure to the day. Taking up or sustaining challenging physical activities in older age can further build self-esteem through mastery and can directly contribute to a fulfilling older age. Moreover, older adults’ families and wider society often have firm views and expectations about how older adults should look and behave. These assumptions may be challenged by those seeking a sense of fun and enjoyment in later life, and physical activities are often the mechanism by which these sensations may be realised. Physical activity thus serves as a mediator for a purposeful and engaging older age and should therefore be treated as a means to an end and not as an end in itself. However, given the real and perceived impediments to being more physically active we will need to restructure our social and physical environments to facilitate individuals and communities to create this sense of purpose and engage in more physical activity.

## Discussion

### Principal findings in context

Whilst many physical activity messages to older adults focus largely on the protective health benefits [74], our findings suggest that interventions may have more traction with older adults if the emphasis shifts to how physical activity contributes to a purposeful and fulfilling life. We suggest that whilst older adults are aware of the health benefits of being active, there are other drivers for behaviour in older age which may be more powerful as behaviour change levers, such as desiring a sense of purpose and feeling needed by and connected to others.

We present an interpretive qualitative analysis that is theory-generating only; further empirical work would be required to explore the validity of the theoretical constructs presented. However, the findings of our meta-ethnography are aligned with, and complement, existing theories of ageing and of behaviour change.

The classical theories of ageing include the activity theory and social breakdown theory. Activity theory asserts that successful ageing occurs only when adults remain active and socially engaged as they age [75]. The findings of the meta-ethnography largely support the main tenets of activity theory in that older adults attain satisfaction from overcoming challenges and adapting to a life after the loss of previous roles. However, rather than adapting to pursue alternative activities, activity theory proposes that this life satisfaction is achieved by older adults maintaining the activities and lifestyle they had in their younger years. In contrast to this latter point, our findings suggest that lifelong physical activity patterns, whilst helpful, are not necessary and new activities taken up in later life may also lead to life satisfaction. Social breakdown theory [76] reasons that older adults suffer from a loss of identity when their social “labels” are removed, as happens in retirement, with the death of a spouse, or when a caregiving role becomes redundant. Subsequent effects on sense of self-worth and identity may explain why physical activity is not readily taken up in this population. This resonates with the third-order constructs “how physical activity fits in with transition events in ageing” and “the role of physical activity in older adults’ sense of purpose and self-belief” [76]. The importance in halting the loss of identity and reconstructing a sense self-worth – through creating challenge and purpose in older age – is supported by the third-order constructs that emerged from the meta-ethnography proposing that physical activities can provide this sense of purpose.

A psychological model of aging, selective optimisation with compensation [77], is also relevant to our findings as this theory – that older adults purposively select activities that are feasible given their physical limitations – can be seen as a necessary approach for older adults in successfully reaching new physical activity goals and achieving a sense of competence. Moreover Carstensen’s socioemotional selectivity theory [78] suggests that older adults develop a preference for social contact with familiar individuals as they age, as these relationships are rewarding and satisfying. This is consistent with our third-order construct on togetherness and belonging as social contact through physical activity is likely to involve familiarity and will contribute to the sense of being needed and missed by the group when absent from the activity. Our findings can also be viewed as complementary to Gullette’s sociological discourse on ageing, which describes how Western society has adopted a cultural meaning of ageing as a period of decline. Gullette introduces the terms “progress narrative” and “decline narrative” to describe how society views early and later life respectively [79]. Our findings suggest that physical activity may provide a means by which older adults can turn the negative “decline narrative” into a continued “progress narrative”, despite major transition events such as physical changes, new diagnoses, and changing life circumstances.

Many behavioural interventions are based on classic theories such as the health belief model [80], social cognitive theory [81], and theory of planned behaviour [82]. There are elements of unity between our findings and components of these theories – for example the probability of physical activity providing a sense of purpose may be viewed as an “expectancy” in social learning theory and the sense of mastery obtained through physical activity could be seen to affect self-efficacy within the health belief model. However, the findings of our meta-ethnography are not wholly consistent with any classic behavioural change theories. Indeed, recent academic discourse has thrown doubt on the value of at least one of these classic theories in changing behaviour in the real world [83]. On the other hand, the findings of this study are harmonious with a relatively new theory of behaviour change, self-determination theory [84], which purports that for behaviour change to occur an individual’s three core psychological needs must be met: the need for competence; the need for relatedness; and the need for autonomy. Whilst self-determination theory is not specific to ageing, these three psychological needs can be viewed as consistent with the third-order constructs retrieved from our synthesis: where physical activity provides a sense of purpose, personal achievement, and self-belief; where physical activity contributes to a sense of togetherness and belonging; and where physical activity routines give a sense of personal responsibility.

### Strengths

This is the first time, to our knowledge, that qualitative literature on physical activity and ageing has been brought together and synthesised using meta-ethnography. Our findings resonate with elements of other theories however this synthesis provides unity of theory, relating specifically to physical activity in ageing. A second key strength of this review is the use of the meta-ethnographic method to re-conceptualise the subject, whilst remaining empirically grounded in the second-order constructs from the constituent papers. As an interpretive synthesis of the primary research, our findings provide different insights to the concepts published in other qualitative syntheses [19–21, 23]. Whilst we describe a new overarching way of considering physical activity in older adults, elements of our findings have been reported by others, for examples in a recent review of studies of physical activity interventions for older adults the authors concluded that engagement may be increased by presenting interventions as fun, sociable, and achievable [21]. This conclusion supports one of the third-order constructs in our synthesis: the value of physical activity in creating feelings of pleasure and wellbeing.

The reliability of a systematic review is related to the quality of its component studies. Whilst we recognise that some view quality assessment of qualitative research as controversial from a relativist position, it can also be argued that if the findings of qualitative syntheses are to be used to influence health and public health policy, then we must be sufficiently confident that the findings of the individual papers making up the synthesis are trustworthy. Our evaluation of study quality using an appropriate framework, attempting to ensure that the included findings have integrity and a degree of dependability, is therefore a further strength of this study.

### Limitations

Despite a comprehensive and updated literature search, it is always possible that some important studies were not identified, and key constructs therefore not analysed. The exclusion of studies not published in English is a limitation of this study, and the small contribution of research from upper middle-income countries means the findings might not be generalizable to all populations. Whilst these papers were readily synthesisable with those from high-income countries it is possible that if the sample of papers from non-high-income countries had been larger, greater heterogeneity may have emerged. Furthermore, with globalisation and the epidemiological transition in lower income countries, the prevalence of non-communicable diseases - and associated risk factors including low physical activity levels – increases [85]. It is therefore possible that the findings will become increasingly applicable to many lower-income countries however it is important to test this and more primary research in those countries would be welcomed. Further high-quality, primary qualitative research on physical activity and ageing from non-high-income countries would enable the meta-ethnography to be updated to include a greater diversity of settings.

It was not possible to comment on whether there are differences by gender as only a small number of studies involved female-only populations (and none male-only), the original primary papers did not provide findings stratified by gender, nor did they include sufficient detail on whether gender was an important conceptual differentiator.

In addition, meta-ethnography, as an interpretivist approach, will be influenced to some degree by the authors’ own experiences, perspectives, and preconceptions. To reduce undue influence over the data interpretation and theory-building, a transparent, referenced, and fully-documented analytical methodology was employed, and emergent constructs were discussed and checked amongst the study team.

We were unable to quantify the physical activity levels of the participants included in the synthesised papers. Our eligibility criteria ensured that “master athletes”- those older adults competing at elite levels - were not included in the synthesis, yet it is possible that the level of physical activity and fitness still varied considerably amongst participants in the included studies. Nevertheless, the constructs derived from these studies should be relevant to older adults across the activity spectrum, and the heterogeneity of the study populations is likely to mirror the heterogeneity of the wider older adult population.

### Implications and unanswered questions

Increasing physical activity levels amongst adults of all ages, including those aged over 65, is a public health priority. In the UK, current National Health Service (NHS) guidelines recommend adults over 65 who are generally fit with no health conditions should undertake at least 150 min of moderate aerobic activity, for example cycling or walking, every week. The latest self-report data published by the Health Survey for England in 2016 reveal that less than half (44%) of the population aged over 65 are meeting this target and the proportion reduces further for those aged 75 years and older (30%) [86]. Our study adds value to the field as our findings provide a plausible explanation for why physical activity levels amongst older adults remain low despite investment in public health programmes and interventions.

The findings presented in this synthesis suggest that physical activity should be framed as a mechanism for supporting a purposeful, socially-connected, and usefully engaged life in older age. As such it provides public health practitioners and gerontologists with an approach, grounded in qualitative data, upon which new programmes and interventions can be developed. Our findings may be used to develop or enhance interventions at the level of the individual, community, or population by reframing the focus to better meet older adults’ motivations. Indeed, a recent paper using qualitative methods to develop a typology of older adults’ motivations for physical activity also included reference to those seeking “purposeful” activity [87].

Further work is required to explore how interventions can incorporate the constructs presented in this paper. An approach moving away from the paternalistic “physical activity for health” message is necessary yet replacing this with a paternalistic “physical activity for purpose” message is unlikely to be effective. Public health specialists will need to create environments in which older adults can both recognise the value of a physically active lifestyle and be enabled to change their behaviour to receive these wider benefits. Age-friendly cities and communities [88] provide a useful framework within which public health specialists and academics can develop and implement physical activity interventions. Comprehensive evaluation of such interventions will provide better understanding of how the third-order constructs identified in this synthesis can effect change at an individual and population level and will lead to the delivery of more effective and sustainable programmes to improve physical activity levels amongst older adults in the longer term.

## Conclusions

Existing physical activity interventions for older adults often emphasise the health benefits as a means of encouraging behaviour change. This meta-ethnography suggests that for older adults, such a focus may be misplaced because at this stage in their lives older adults have other goals which are of greater personal importance. Physical activity however can be a means by which these goals may be obtained. We therefore suggest that for an intervention to effectively increase and sustain physical activity levels in older adults a shift in focus is required. We advocate an approach in which older adults are supported to regain or consolidate their sense of purpose through the routines, personal achievement, and meaningful social connections physical activity can provide.

## Additional file

Additional file 1: **Table S1.** Description of papers included in meta-ethnography. (DOCX 20 kb)

## Abbreviations

NHS: National Health Service
PRISMA: Preferred Reporting Items for Systematic Reviews and Meta-Analyses
RCT: Randomised controlled trial
UK: United Kingdom
US: United States
