# Supplementary material for: A life fulfilled: positively influencing physical activity in older adults – a systematic review and meta-ethnography
Source: BMC Public Health. 2019 Apr 2;19:362. doi: 10.1186/s12889-019-6624-5 (PMC6444855; doi:10.1186/s12889-019-6624-5)
Supplement: Supplementary file 1 — Table S1. Description of papers included in meta-ethnography. (DOCX 20 kb) [file 12889_2019_6624_MOESM1_ESM.docx]

Supplementary Table 1 Description of papers

| **Year** | **First author** | **Gender** | **Setting** | **Ethnicity / Faith / Culture** | **Purpose / aim** | **Study design** | **Sample size** | **Age (y)** |
| --- | --- | --- | --- | --- | --- | --- | --- | --- |
| 1995 | Duncan | Mixed | US | Caucasian | Explore motivation for mall walking in older adults | Observations; interviews | 14 | 60+ |
| 2001 | Lavizzo-Mourey | Mixed | US | African American | Develop a PA intervention in an urban community of African Americans | Focus groups | 38 | 65+ |
| 2002 | Cooper | Mixed | UK | None specified | Explore meaning of social dancing for older people | Ethnography; interviews | 6 sites; 31 interviews | 60+ |
| 2003 | Grossman | Mixed | US | None specified | Explore perceptions about PA in inactive older adults | Semi-structured interviews | 33 | 75+ |
| 2004 | Stathi | Mixed | UK | None specified | Understand PA and ageing in people in exercise referral schemes | Semi-structured interviews | 13 | 63-79 |
| 2005 | Dumas | Mixed | US | Caucasian | Explore motivation for mall walking in older adults | Observations; interviews | 14 | 60+ |
| 2005 | Paulson | Mixed | UK | None specified | Explore how PA contexts shape ageing experience | Ethnography; interviews | 2 sites | 50+ |
| 2005 | Rydeskog | Mixed | Sweden | None specified | Describe elderly people’s own experiences of resistance training | Semi-structured interviews | 15 | 63-87 |
| 2005 | Wagstaff | Female | US | None specified | Explore elders’ perspectives about PA participation | Semi-structured interviews | 5 | 75-94 |
| 2007 | Witcher | Mixed | Canada | None specified | Assess rural older adults’ perceptions of PA | Semi-structured interviews | 10 | 70-94 |
| 2008 | Grant | Mixed | NZ | Caucasian | Explore meanings about experiences when increasing PA | Semi-structured interviews | 26 | 70+ |
| 2009 | Bidonde | Female | US | None specified | Describe the meaning of group fitness to older women living alone | Semi-structured interviews; artefact documentation | 9 | 67-83 |
| 2009 | Jancey | Mixed | Aus | None specified | Identify issues and perceptions about PA in older adults | Semi-structured interviews | 16 | 65-74 |
| 2010 | Beck | Mixed | US | None specified | Examine impact of retirement on PA | Semi-structured interviews | 11 | 57 – 65 |
| 2010 | Grant | Mixed | Canada | None specified | Examine experiences about walking in neighbourhoods | Focus groups; interviews | 75 | 65+ |
| 2010 | Horne_a | Mixed | UK | White; South Asian | Explore influence of PCHCPs in increasing PA in older adults | Focus groups; Semi-structured interviews | 125 | 60-70 |
| 2010 | Leavy | Mixed | Stockholm; Dublin | Swedish; Irish | Describe perceptions of PA held by older urban Swedish and Irish adults | Semi-structured interviews | 30 | 65+ |
| 2011 | Abolfazl | Mixed | Iran | Shiite Muslims | Explore experiences of exercise for elderly Iranians | Semi-structured interviews | 16 | 65 – 85 |
| 2011 | Costello | Mixed | US | None specified | Understand beliefs about PA in independent older adults | Focus groups | 31 | 60+ |
| 2011 | Henwood | Mixed | Aus | None specified | Study benefits attributed to resistance training by older adults | Focus groups | 18 | 65-81 |
| 2012 | Barnett_a | Mixed | n/a | None specified | Understand experiences of transition to retirement | Systematic review and narrative synthesis of quant and qual | 5 qualitative studies | 50 – 94 |
| 2012 | Bjornsdottir | Female | US | None specified | Understand PA experiences of older women in retirement communities | Semi-structured interviews | 10 | 70 or older |
| 2012 | Horne_b | Mixed | UK | South Asian | Identify the attitudes and beliefs associated with the uptake and adherence of PA among community-dwelling South Asians | Focus groups and in-depth interviews | 46 | 60 – 70 |
| 2012 | Liu | Mixed | UK | Chinese | Explore behaviours and attitudes towards exercise among older Chinese immigrants in the UK | Semi-structured interviews | 33 | 60 – 84 |
| 2012 | Lubcke | Mixed | Sweden | None specified | Investigate what factors influenced older adults to start to continue to exercise in a senior gym | Semi-structured interviews | 8 | 65 – 81 |
| 2012 | Welmer | Mixed | Sweden | None specified | Describe experiences of PA, perceived meaning, and the importance of and motives for participation in PA | Focus groups | 20 | 80 – 91 |
| 2013 | Barnett_b | Mixed | UK | None specified | Explore and describe how couples influence each other’s PA behaviour in retirement | Semi-structured interviews with couples | 14 (7 couples) | 63 – 70 |
| 2013 | Franke | Mixed | Canada | None specified | Examine key factors that facilitate PA in highly active community-dwelling older adults | In-depth interviews | 10 | 65 – 88 |
| 2013 | Horne_c | Mixed | UK | South Asian and White British | Explore barriers to initiating and maintaining regular PA among UK Indians, Pakistani, and White British adults | Focus groups and in-depth interviews | 15 focus groups; 40 interviews | 60 – 70 |
| 2013 | Li | Mixed | China | Chinese | Explore the experiences and perceptions of the elderly community regarding physical activity and to gain a better understanding of these | Semi-structured interviews | 12 | 63-82 |
| 2013 | Price | Female | US | Black | Examine what contributed to their PA initiation and maintenance | Semi-structured interviews | 15 | 60+ |
| 2014 | Phoenix | Mixed | UK | None specified | Research and theorize the concept of pleasure in relation to PA | Life history interviews and photoelicitation | 51 | 60 – 95 |
| 2015 | Alizadeh | Mixed | Iran | Iranian | Explore the perspectives of Iranian elders regarding health, healthy eating, and PA | Focus groups | 60 | 60 – 97 |
| 2015 | Franco | Mixed |  | None specified | Identify and synthesise the range of barriers and facilitators to PA participation | Systematic review and thematic / content analysis | 132 qualitative studies | 60+ |
| 2015 | Kenter | Mixed | Netherlands | None specified | Explore how life events influence PA patterns of older adults | Life history interviews | 17 | 60 – 82 |
| 2015 | McDonald | Mixed | UK | None specified | Explore and compare perceptions about how theory-based factors influence PA change during the transition from employment to retirement | Semi-structured interviews | 28 | 55 – 67 |
| 2015 | Sebastiao | Female | US | African American | Explore perceptions of PA among African American women and the socio-cultural factors that influence their participation | Semi-structured interviews and photoelicitation | 20 | 60 – 80 |
| 2016 | Guell | Mixed | UK | None specified | Describe and explore perceptions, practices, and motivations for active living in later life | Semi-structured interviews and participant observations | 27 | 65 – 80 |
| 2016 | Witcher | Mixed | Canada | Rural settings | Identify factors that influenced PA participation and explore role of rural context | Semi-structured interviews | 20 | 68 – 97 |
